# Cost of implementing evidence-based practices to reduce opioid overdose fatalities in New York State communities

**DOI:** 10.1186/s13722-025-00606-6

**Published:** 2025-10-01

**Authors:** Jazmine M. Li, Dawn Gruss, Timothy Hunt, James David, Emma Rodgers, Nabila El-Bassel, Bruce R. Schackman, Laura E. Starbird

**Affiliations:** 1 Department of Population Health Sciences, Weill Cornell Medicine, 575 Lexington Avenue, New York, NY 10022 USA; 2 Social Intervention Group, Columbia University School of Social Work, 1255 Amsterdam Ave, New York, NY 10027 USA; 3 National Center for Engagement in Diabetes Research, NYU Langone Health, 550 1st Ave, New York, NY 10016 USA; 4 Department of Family and Community Health, School of Nursing, University of Pennsylvania, 418 Curie Blvd, Philadelphia, PA 19104 USA

**Keywords:** Cost analysis, Opioid use disorder, Community engagement, Evidence-based practices, HEALing communities study

## Abstract

**Background:**

The HEALing Communities Study was a multi-site cluster randomized waitlist-controlled trial evaluating a community-engaged, data-driven intervention to select and deploy evidence-based practices (EBPs) including overdose education and naloxone distribution (OEND), medication for opioid use disorder (MOUD), and safer opioid prescribing. The trial was conducted in 67 highly impacted communities in 4 states, including 8 Rural and 8 urban communities in New York State (NYS). To inform future community-level decision making, we estimated the implementation costs of the EBPs selected by NYS communities.

**Methods:**

The study was implemented between January 2020-June 2022 (Wave 1, 30 months duration including the peak COVID-19 emergency period) and July 2022-December 2023 (Wave 2, 18 months); each wave included 4 Rural and 4 urban NYS communities. We collected cost data prospectively using invoices, administrative records, and interviews with program staff and stakeholders. We then conducted a micro-costing analysis from the community perspective and compared costs from Waves 1 and 2.

**Results:**

In both Waves, each community deployed on average 15 EBPs (range 8–25). EBP costs averaged $705,000 (range $320,000-$1.3 million) and $312,000 (range $39,200-$686,300) in Waves 1 and 2, respectively. In Wave 1, 25% of costs were allocated for OEND, 71% for MOUD, and 4% for safer prescribing, compared to 38% for OEND, 60% for MOUD, and 2% for safer prescribing in Wave 2. Average EBP costs per community were $147,600 (range $20,900-$374,000) for those in the OEND category, $345,400 (range $4,100-$1.1 million) for MOUD, and $16,400 (range $360-$105,500) for safer prescribing. Total EBP cost per capita in urban communities was $0.32 compared to $2.65 in Rural communities in Wave 1, and $0.41 urban communities compared to $0.65 in Rural communities in Wave 2.

**Conclusions:**

The lower EBP costs in Wave 2 resulted from differences in EBP categories and specific EBPs selected and may also reflect differences in the duration of the intervention and the impact of the COVID-19 pandemic over time. Higher per capita costs in rural communities indicate that many costs were not directly related to the number of individuals served.

**Supplementary Information:**

The online version contains supplementary material available at 10.1186/s13722-025-00606-6.

## Introduction

Drug-related overdose deaths have risen in the United States (U.S.) over the past 2 decades [1]. Among people in the U.S. aged 12 or older in 2022, 8.9 million (3%) misused opioids within the past year, and there were an estimated 81,083 fatal opioid overdoses in 2023 [2]. Despite recent positive trends in opioid overdose fatalities [3], there is a critical need for community-engaged, evidence-based interventions to address the opioid crisis.

The HEALing (Helping to End Addiction Long-term) Communities Study was a community-engaged, multi-site study to examine the efficacy of the Communities That HEAL intervention to reduce opioid-related overdose deaths through the implementation of an integrated set of evidence-based practices (EBPs) in health care, behavioral health, justice, and community-based settings [4, 5]. The intervention was implemented in 67 highly-impacted Rural and urban counties and townships across four U.S. states; 16 communities, including 13 counties and three communities comprised of specific townships or cities, participated in New York State (NYS). The protocol, community engagement process, and trial results have been described in detail previously [5–10].

Few studies have examined the resources required to implement large-scale, community-driven interventions to address the opioid crisis. Cost-benefit analyses of the Communities that Care model, a substance use prevention intervention for adolescents, indicated that it was a cost-beneficial approach with a conservative cost estimate of $991 per youth and net present benefit of $4,259 per youth over 5 years [11], and that economic gains in youth were confirmed in analyses incorporating outcomes over the subsequent three years [12]. These results show that a community-based substance use prevention model focused on youth can have economic benefits. However, to our knowledge the economic costs of implementing multiple broad community-level EBPs with adults to reduce opioid overdoses have not been widely evaluated or reported. The objective of this study was to estimate the cost of the resources used to implement the EBPs selected by each of the 16 communities participating in the HEALing Communities Study in New York State and to compare costs to communities that implemented the intervention in two different time periods.

## Methods

This study protocol (Pro00038088) was approved by Advarra Inc., the HEALing Communities Study single Institutional Review Board (sIRB).

### Overview of the intervention

The intervention supported coalitions in each community to select and implement a set of EBPs to reduce opioid overdose fatalities. Coalitions, made up of stakeholders representing diverse sectors such as healthcare and mental health providers, emergency responders, law enforcement, substance use treatment providers, and persons with lived experience, used a data-driven process to select EBPs tailored to their community in the following categories: (1) opioid overdose prevention and naloxone distribution (OEND) in high-risk populations, (2) effective delivery of medication for opioid use disorder (MOUD) maintenance and treatment, including agonists/partial agonists medication and outreach and delivery to high-risk populations, and (3) safer opioid prescribing and dispensing [5]. The above categories were further broken down into Passive and Active OEND, MOUD linkage, MOUD engagement and retention, and MOUD expansion. Passive OEND is defined as, “overdose prevention and response education and naloxone rescue kit distribution to people referred by other care providers or for those seeking OEND on their own,” while Active OEND is defined as, “proactive distribution of overdose prevention and response education and naloxone rescue kits to higher risk populations and their social networks” [13]. Communities were required to implement a minimum of 5 strategies, with at least one in the OEND category, three in the MOUD category, and one in the safer prescribing category. EBPs were selected using the Opioid-overdose Reduction Continuum of Care Approach (ORCCA) [13], which consisted of a menu that outlined strategies and supporting research, example milestones and measures, and resources and toolkits for each required category. Other elements of the study, including the community engagement process itself and communication campaigns to increase awareness and demands for EBPs and to reduce stigma, are described elsewhere [8] and are not examined here, as our focus is on the costs of implementing the EBPs that were selected. EBPs were considered to have been implemented if at least one person received that EBP service. The duration of EBPs varied by community and EBP; for example, the longest EBP lasted 12 months and the shortest EBP lasted 3 months.

The HEALing Communities Study was implemented in NYS from January 2020-June 2022 (Wave 1, 30 months duration including the peak COVID-19 emergency period; the duration was increased from 24 months to 30 months in December 2020) and July 2022-December 2023 (Wave 2, 18 months). Each wave included 4 Rural and 4 urban communities, for a total of 16 communities. Communities were classified as rural or urban using National Center for Health Statistics criteria, where “metropolitan” communities were considered urban and “nonmetropolitan” communities were considered rural [14]. We estimated the implementation costs, excluding research-specific costs, for each EBP selected from the ORCCA menu by all 16 communities. Costs included those that were reimbursed by HEALing Communities Study according to a predetermined budget that varied by community, other costs incurred by the community but not reimbursed by the HEALing Communities Study, and non-HEALing Communities Study grants that were received by the community. We used invoices and records of funds received from other grants, complemented by an activity-based micro-costing approach [15] from the community perspective where appropriate [10, 16]. 

### Cost data collected from invoices and grants

Community EBP budgets were determined by a funding algorithm that considered population size, as well as contextual factors including fatal overdose rate, emergency department visit and hospitalization rates, and jail census. Wave 1 communities also had funds added due to the extension of the intervention period during the peak COVID-19 emergency period. Purchases to support EBP implementation, such as vehicles, naloxone housing units, laptops, tablets, trainings, and media printing were primarily captured via invoices submitted by the communities to the HEALing Communities Study for reimbursement. Other invoiced costs included those incurred as a result of contracts with external non-governmental partners, such as substance use treatment and harm reduction organizations, that carried out EBP implementation for a fee or invoiced for salary and fringe costs for their staff time to support EBPs. Invoice costs were typically allocated to a specific EBP by the community prior to submitting to the HEALing Communities Study for reimbursement. Where an invoice did not directly allocate expenses to a specific EBP, research staff reviewed all receipts accompanying the invoice for labels or indicators of which EBP it aligned with and verified assumptions with HEALing Communities Study staff who had knowledge of the community’s spending.

We included grant funds received by the communities from non-HEALing Communities Study entities if they directly supported newly implemented EBPs. We used study records documenting these grants in addition to interviews with HEALing Communities Study staff to allocate these additional grant funds to specific EBPs and avoid double-counting costs we had previously identified elsewhere. Grant funds that could not clearly be allocated to a specific EBP were excluded.

### Microcosting other costs

We conducted interviews with program management staff in each community directly supported by HEALing Communities Study funds to oversee EBP implementation to clarify, expand on, and capture costs not accounted for in invoices. Where necessary, research staff then followed up with semi-structured interviews with partner organizations that were selected to carry out specific EBP activities and community liaisons who supported EBP implementation. Interviews followed a structured process and interview guide developed by the HEALing Communities Study Consortium [14].

Research staff conducted at least two interviews with each Program Manager in Wave 1 communities; this was streamlined into one interview for each Wave 2 community based on lessons learned from Wave 1, for a total of 25 program management staff interviews and 14 partner organization interviews across both Waves. In both Waves, we conducted follow up over emails and telephone. We also reviewed administrative records for each community, including implementation plan agreements with community partners and monitoring forms that tracked activities for each EBP. These additional records helped us understand the EBP implementation process and identify additional individuals or entities that played a role in implementation.

We multiplied nationally representative wage and fringe rates from the Bureau of Labor Statistics (BLS) [17] by time estimates collected in interviews (Supplementary Material 1, S1). We also included estimated cost of resources, such as bags to hold naloxone and other rescue supplies that were assembled by county staff and not included in invoices, as well as the time spent by county staff assembling the bags. We did not include the cost of naloxone itself, since naloxone is provided by NYS Department of Health [18] to the community organizations and local governments without charge. We separated one-time costs from ongoing monthly costs and assigned the ongoing costs for the time period for which the EBP was implemented (Supplementary Material 1, S2).

### Costs summarized by community and secondary analysis

For each EBP in each community, we summed costs collected from invoices or external grants and costs determined by micro-costing [15]. Micro-costing is a bottom-up approach using detailed data on each element of an intervention by tracking and valuing every resource used (See Supplementary Material 1, S2). For any EBPs that had missing data, we made time or cost assumptions using estimates from communities that implemented a similar EBP. This occurred for 9 of 239 EBPs.

The cost of staff directly supported by HEALing Communities Study funds in the communities (in most cases, a Program Manager, Data Coordinator, and Community Engagement Facilitator) was captured separately. Data collected quarterly for these staff members included wage, fringe rate, and proportion of working hours spent on HEALing Communities Study activities. These costs are reported separately and not included in our primary analysis of EBP implementation costs because it was not clear how to allocate them to EBP implementation compared to other HEALing Communities Study-related activities, such as managing the community engagement process and communications campaigns. However, to avoid underestimating the total resources required to implement the selected EBPs in each county, we conducted a secondary analysis that includes these costs, which may represent the upper bound of costs.

## Results

We excluded any no-cost EBPs from the total number of EBPs and this resulted in a total of 183 EBPs for which primary EBP cost results are reported. Of the 239 total EBPs selected by communities, 56 (23%) EBPs had no incremental cost (i.e., there were no additional activities that incurred any cost beyond what a community was already incurring prior to the study), 22 (9%) were discontinued prior to implementation (4 in Wave 1 and 18 in Wave 2), 23 (10%) only included costs of HEALing Communities staff time (captured in secondary analyses), 6 (3%) were carried out solely by organizations outside of the HEALing Communities Study and their costs were captured elsewhere as part of the community engagement process for the study, 3 (1%) were unable to be costed due to lack of information, and 2 (0.8%) were combined with another EBP (Supplementary Material 1, S4). Costs reported in the interviews largely consisted of unpaid time to implement the EBPs and additional in-kind purchases that were not reimbursed.

An average of 15 EBPs were implemented by each community in both Waves 1 and 2 (Wave 1 range 8–25; Wave 2 range 11–22). In Wave 1, 25% of EBP costs were for OEND, 71% for MOUD, and 4% for safer prescribing, compared to 38% for OEND, 60% for MOUD, and 2% for safer prescribing in Wave 2. MOUD represented more than half of costs in 7 of 8 Wave 1 communities versus 6 of 8 Wave 2 communities.

**Fig. 1:**
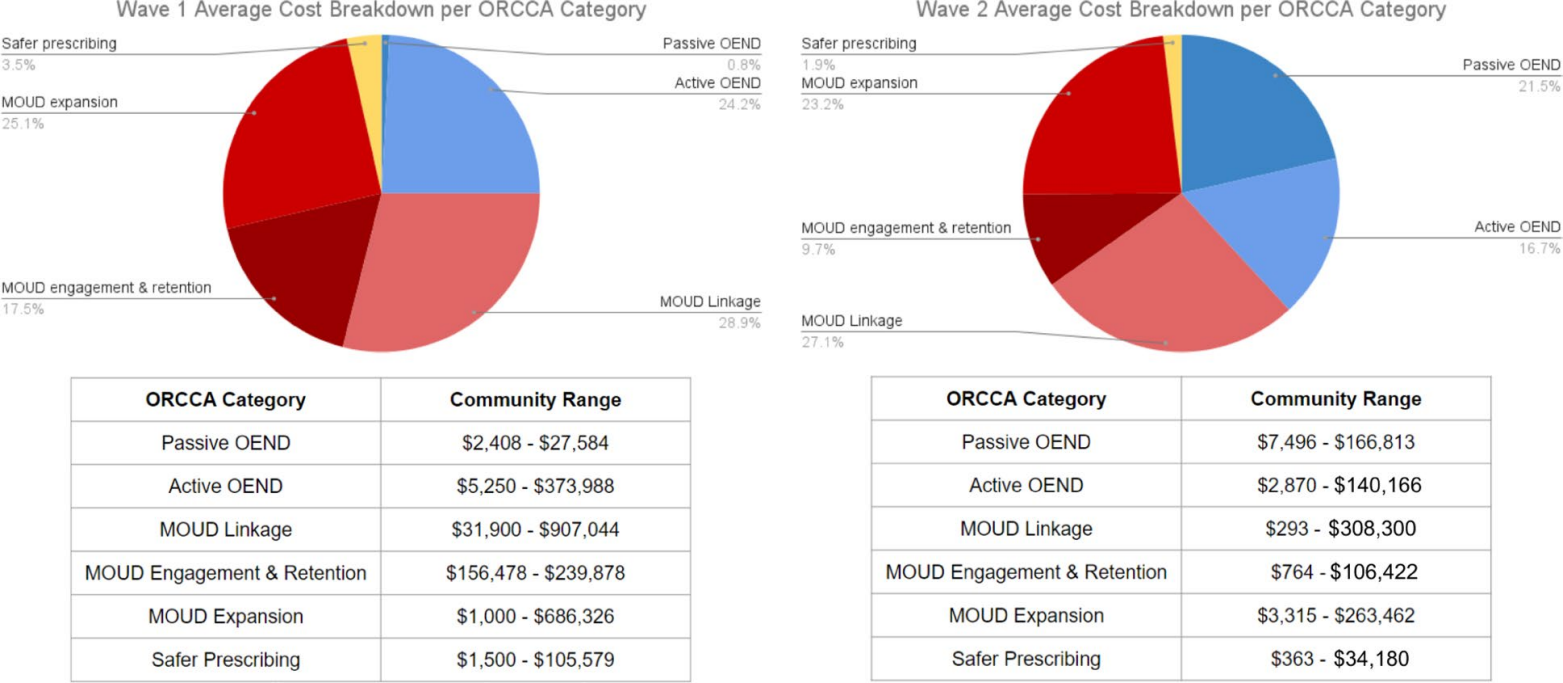
Cost Breakdown by Category of Opioid Strategy by Community Study, Waves 1 and 2. OEND, overdose education and naloxone distribution; ORCCA, Opioid-Overdose Reduction Continuum of Care Approach; MOUD, medications for opioid use disorder

Wave 1 implemented a greater number of criminal justice-based active OEND strategies, whereas Wave 2 focused largely on passive OEND strategies including partnerships with emergency medical services and naloxone leave-behind initiatives (Fig. 1). Waves 1 and 2 selected similar MOUD strategies including peer referral and linkage to MOUD via correctional facilities, street outreach, and leveraging the NYS Department of Health NY Matters program resources to help initiate care, increase access to medication, and link individuals to treatment [19]. In the safer opioid prescribing/dispensing category, both Waves implemented county-wide training for pharmacists and providers and drug take-back events.

Wave 1 EBPs cost a total of $5,639,800 across all 8 communities with an average cost of $705,000 per community. Wave 2 EBPs cost $2,493,503 across all 8 communities, with an average cost of $311,700 per community (Table 1). The average cost to implement an OEND EBP was lower than the average cost to implement an MOUD EBP, and safer prescribing EBPs had the lowest cost in both Waves. Average EBP costs per community were $147,600 (range $20,900-$374,000) for those in the OEND category, $345,400 (range $4,100-$1.1 million) for MOUD, and $16,400 (range $360-$105,500) for safer prescribing. Average costs were higher in Wave 1 versus Wave 2 for all sub-categories except Passive OEND.

**Table 1:** Average cost to implement an opioid overdose reduction evidence-based practice by category of evidence-based practice

EBP Category	# of Communities that Implemented EBP(s) in Category*		Average Cost of an EBP		Range of EBP Costs	
	Wave 1	Wave 2	Wave 1	Wave 2	Wave 1	Wave 2
Active OEND	8	8	$170,626	$51,913	$5,250 - $373,988	$2,870 - $140,166
Passive OEND	3	8	$15,205	$66,868	$2,408 – $27,584	$7,496 - $166,813
MOUD Linkage	7	7	$233,909	$84,482	$31,900 - $907,044	$328 - $308,300
MOUD Engagement and Retention	5	6	$123,166	$30,188	$194,101 - $239,878	$764 - $106,422
MOUD Expansion	8	7	$176,647	$72,414	$1,000 - $686,326	$3,431 - $263,462
Safer opioid prescribing and dispensing	8	7	$24,904	$5,797	$1,500 - $105,579	$363 - $34,180

*Excludes communities that did not implement any EBPs in the category or only implemented EBPs in the category with $0 cost to the community

EBP, evidence-based practice; OEND, overdose education and naloxone distribution; MOUD, medications for opioid use disorder

EBPs that were frequently selected by the communities in Waves 1 and 2 are shown in Table 2. Pharmacist and provider safer dispensing was the most common low-cost (i.e., under $50,000) EBP, whereas peer navigation for MOUD engagement and retention was a frequently implemented high-cost (e.g., more than $50,000) EBP.

**Table 2:** Cost of frequently implemented evidence-based practices for opioid overdose reduction

EBP Category	Frequently implemented EBP	Number of communities that implemented this EBP		Median cost (range)
		Wave 1	Wave 2	
Safer Prescribing	Pharmacist and/or provider safer opioid dispensing training	6	6	$3,175 ($363-$29,877)
Passive OEND	Naloxone housing units in overdose hotspots	5	8	$37,651 ($863-$156,828)
MOUD engagement and retention	Peer navigation	3	4	$75,528 ($29-$320,094)
Active OEND	First responder naloxone leave-behind	1	5	$15,805 ($181-$38,721)
Active OEND	OEND provided at jail release	3	5	$5,844 ($623 - $26,000)
MOUD expansion	MOUD expansion in jails	1	5	$38,155 ($8,275 - $174,000)
Active OEND	Naloxone distribution via community pop-up events	2	2	$11,071 ($1,100 - $24,639)

EBP, evidence-based practice; OEND, overdose education and naloxone distribution; MOUD, medications for opioid use disorder

**Fig. 2:**
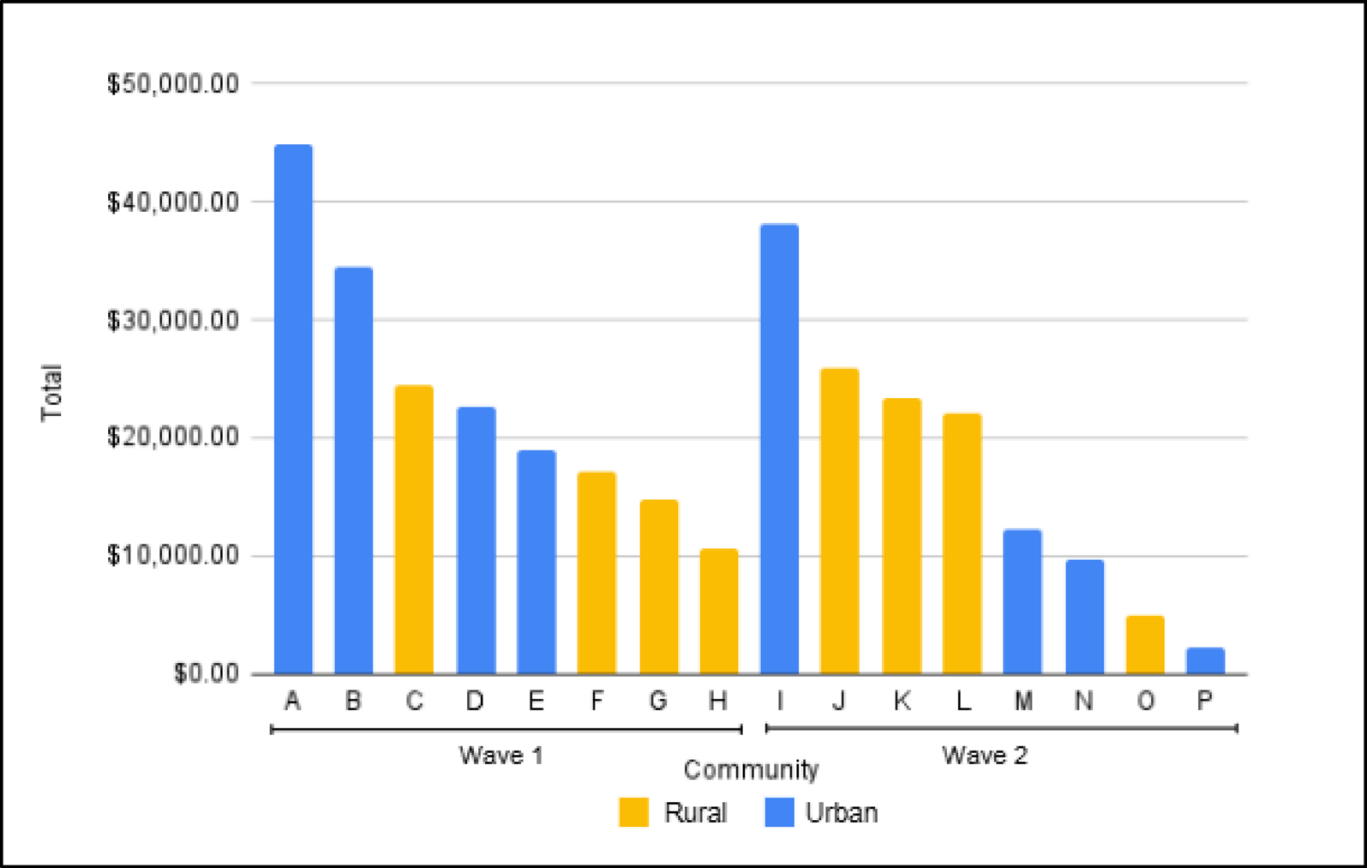
Monthly Spend by Community, Waves 1 and 2

**Fig. 3:**
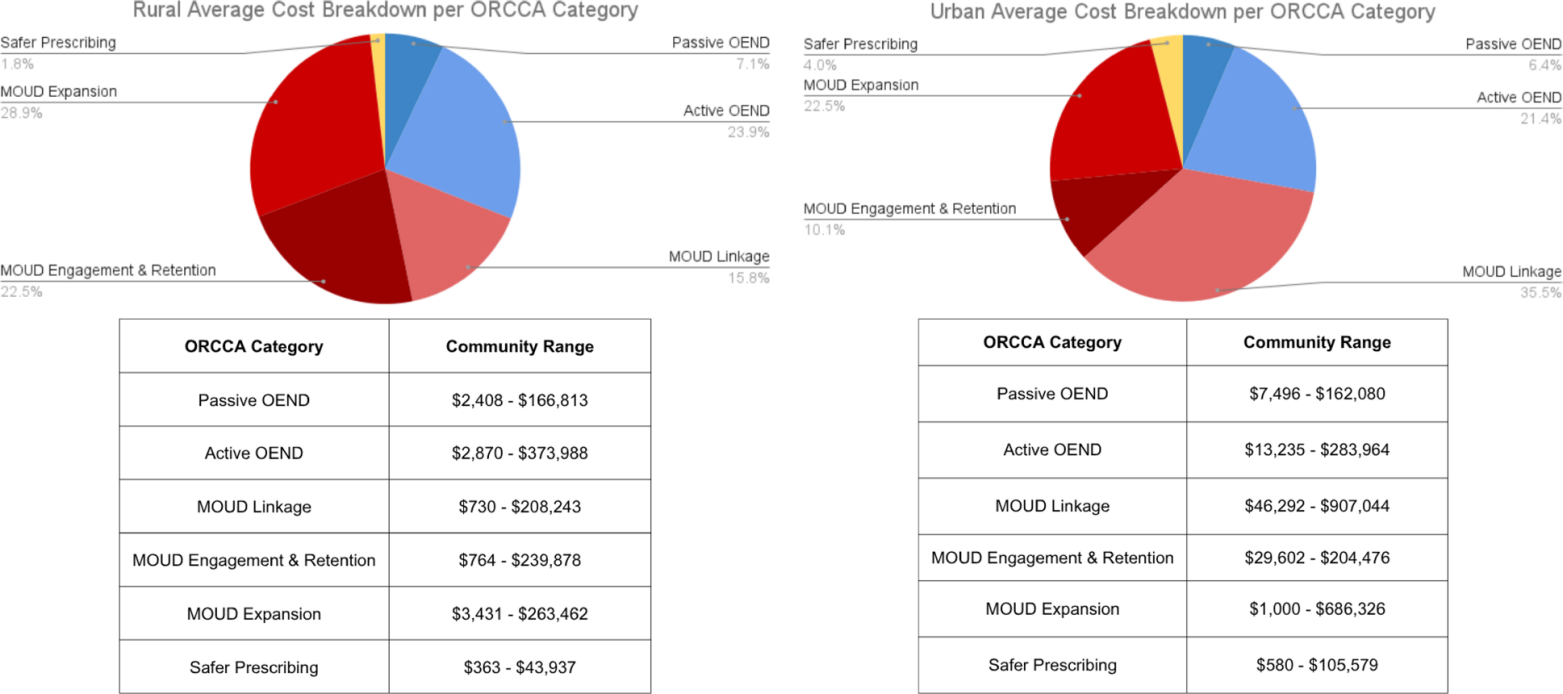
Cost Breakdown by Category of Opioid Strategy by Rural/Urban Designation, Waves 1 and 2. OEND, overdose education and naloxone distribution; ORCCA, Opioid-Overdose Reduction Continuum of Care Approach; MOUD, medications for opioid use disorder

Of the total cost in Wave 1 ($5,639,800), $4,073,229 (72%) was identified in invoices and $1,556,572 (28%) came from micro-costing. Of the total cost in Wave 2 ($2,493,503), $2,061,952 (83%) came from invoices and $431,551 (17%) from micro-costing. The average monthly cost to implement EBPs was about $23,000 in urban communities compared to $17,800 in rural communities (Fig. 2). However, cost per capita was lower in urban communities compared to rural. Total EBP cost per capita in urban communities was $0.32 compared to $2.65 in Rural communities in Wave 1, and $0.41 urban communities compared to $0.65 in Rural communities in Wave 2. Rural and urban communities also differed on their ORCCA spending, with rural communities spending more in the MOUD expansion category. While urban communities spent more towards MOUD linkage (Fig. 3).

While every community had HEALing Communities Study-supported staff, these staff were made up of different combinations of a Program Manager, Data Coordinator, and Community Engagement Facilitator. When the staff time is added to the total EBP costs in each community, Wave 1 EBPs cost a total of $6,743,150 across all 8 communities with an average cost of $842,894 per community. Wave 2 EBPs costs $4,340,430 across all 8 communities, with an average cost of $542,554 per community (Supplementary Material 1, S3).

## Discussion

We estimated the costs of implementing a wide range of opioid overdose reduction EBPs across 16 different communities in New York State. This is the first detailed examination of large-scale substance use intervention costs in multiple communities from a community perspective. The results indicate what communities saw as the most pressing strategies requiring new resources and demonstrate the costs of implementing them across a wide range of communities. The ORCCA menu combines a wide range of EBPs, and demonstrates how overdose reduction strategies work best together and overlap across a continuum of care [8].

There were substantial differences in how communities chose to allocate EBP resources, which may reflect differences in perceived need, feasibility, or other factors. Wave 2 communities on average spent a greater proportion of resources on passive OEND than Wave 1, whereas Wave 1 communities spent greater proportions on MOUD engagement and retention and MOUD expansion. Computer simulation modeling studies of community-level overdose interventions have shown that expanding active OEND in combination with greater MOUD initiation and retention has the greatest potential to achieve sustained reductions in opioid overdose deaths [18, 19].

Costs were substantially higher in Wave 1 compared to Wave 2. Wave 1 communities spent more on average in each EBP category, except for Passive OEND. A greater proportion of EBPs were for MOUD in Wave 1 compared to Wave 2, and MOUD EBPs were on average more expensive than OEND EBPs. Wave 2 communities may have been motivated to spend their funds on OEND strategies that were faster and less expensive to implement due to the shorter time period (18 months) that Wave 2 had to implement the intervention. Conversely, with 30 months to select and implement EBPs in Wave 1, these communities may have chosen strategies that are more time and resource-intensive to implement, particularly in the MOUD categories. Another important difference between the two Waves was the role of HEALing Communities Study-supported local staff. We believe that Wave 2 relied more heavily on those staff members to carry out EBPs, which may have led to lower invoiced and micro-costed costs. This is reflected in the secondary analysis results where the difference in average costs per community between Wave 1 and Wave 2 narrowed. While Waves 1 and 2 are only comparable within the context of these key differences, both approaches contribute to the range of potential costs a community might incur in a real-world scenario.

Considering their larger populations, it is not surprising that total costs were generally higher in urban communities compared to Rural communities in both Waves 1 and 2. However, per capita costs were higher in rural communities in both Waves. Rural residents face additional barriers to accessing care compared to urban residents, including having to travel longer distances to obtain care, having less comprehensive insurance coverage, and having fewer available healthcare providers [20, 21].

### Limitations

Interpretation of our cost analysis may be limited by the availability of data and recall bias. Our micro-costing methods relied on stakeholder interviews for time estimates and process details, but often stakeholders were involved in multiple EBPs and may have omitted important details, resulting in an underestimation of EBP implementation costs. Additionally, there was considerable effort by HEALing Communities-supported staff within each community to initiate EBPs and monitor progress that was essential to successful implementation. Our secondary analysis that included all effort by HEALing Communities-supported staff within each community helped to adjust for this limitation. The length of time that each EBP was delivered varied among communities in both Waves and Wave 1 communities had a longer intervention period during which they could potentially deliver EBPs; however, the COVID-19 pandemic may have delayed the start-up of EBPs and reduced the efficiency of delivering them in Wave 1 communities. Furthermore, our analyses do not account for the ways in which community decision making and EBP implementation could have been affected by the current or expected availability of opioid settlement funds or additional grants and how this might have differed between Wave 1 and Wave 2 communities. We attempted to minimize some of this effect by including non-HEALing Communities Study grants that directly supported EBP implementation in our analyses. Finally, we did not include the cost of naloxone because naloxone is provided by NYS to communities without charge, whereas in other jurisdictions this cost may be borne by local communities. As of 2024, the cost of over-the-counter naloxone was approximately $45 for 2 doses [22].

## Conclusions

This study provides insight into the real-world resources that communities may need to invest to implement evidence-based community interventions to reduce opioid overdose deaths. Costs will vary depending on type of strategies implemented as well as the context, including urban versus rural and other public health needs such as addressing the COVID-19 emergency. Depending on the needs identified by the community and the amount and duration of available funds, these cost differences might affect the choice of services and allocation of funds. Our study provides insight into how much different strategies cost and what resources are needed for communities to implement the evidence-based community-based practices that they select to address the overdose crisis. The overall cost of the HEALing Communities Study intervention, Communities that HEAL [10], and future cost-effectiveness analyses will allow stakeholders to evaluate the value of implementing this large-scale, community-engaged, data-driven process in their communities.

## Supplementary Information

Supplementary Material 1

## Data Availability

The data that support the findings of this study are available from the corresponding author upon reasonable request.

## Abbreviations

BLS: Bureau of Labor Statistics
CTH: Communities That HEAL
EBP: Evidence based practice
EMS: Emergency Medical Services
HCS: HEALing Communities Study
HEAL: Helping to End Addiction Long-term
IRS: Internal Revenue Services
MOUD: Medications for opioid use disorder
OEND: Overdose education and naloxone distribution
ORCCA: Opioid-overdose Reduction Continuum of Care Approach
OUD: Opioid use disorder
SAMHSA: Substance Abuse and Mental Health Services Administration
