# Supplementary material for: Cost of implementing evidence-based practices to reduce opioid overdose fatalities in New York State communities
Source: Addict Sci Clin Pract. 2025 Oct 1;20:77. doi: 10.1186/s13722-025-00606-6 (PMC12486734; doi:10.1186/s13722-025-00606-6)
Supplement: Supplementary file 1 — Supplementary Material 1 [file 13722_2025_606_MOESM1_ESM.docx]

**Additional File 1**

**S1. Unit Costs for Wage Categories, 2024 US Dollars**

| **Position** | **BLS Category Used** | **Hourly Wage** |
| --- | --- | --- |
| CEO | 11-1011 Chief Executives | $124.47 |
| Clinical Directors | 11-9111 Medical and Health Services Managers | $64.64 |
| Community Health Worker | 21-1094 Community Health Workers | $25.30 |
| EMS | 29-2042 Emergency Medical Technicians | $20.72 |
| Fire Chiefs | 33-1021 First-Line Supervisors of Firefighting and Prevention Workers | $43.63 |
| Firefighters | 33-2011 Firefighters | $29.03 |
| Nurse Practitioners | 29-1171 Nurse Practitioners | $61.78 |
| Patient Navigators | 31-9099 Healthcare Support Workers, All Other | $22.60 |
| Pharmacists | 29-1051 Pharmacists | $64.81 |
| Physician | 29-1229 Physicians, All Other | $119.54 |
| Police Officers | 33-3051 Police and Sheriff's Patrol Officers | $36.80 |
| Probation Officers | 21-1092 Probation Officers and Correctional Treatment Specialists | $39.44 |
| Program Directors | 11-1021 General and Operations Managers | $62.18 |
| Public Health Educators | 21-1091 Health Education Specialists | $33.55 |
| Sheriffs | 33-1012 First-Line Supervisors of Police and Detectives | $50.96 |
| Social Workers | 21-1023 Mental Health and Substance Abuse Social Workers | $30.71 |
| Program Managers | HCS Staff Positions | $37.45 |
| Data Coordinators | HCS Staff Positions | $37.23 |
| Community Engagement Facilitators | HCS Staff Positions | $36.29 |

Note: BLS, Bureau of Labor Statistics; hourly wages are the national average; CEO, Chief Executive Officer; EMS, Emergency Medical Services; HCS, HEALing Communities Study

**S2. Example of Micro-costing Process in a Wave 2 Community**

We conducted a micro-costing analysis for emergency medical service (EMS) leave-behind naloxone initiatives in multiple communities. One-time costs to prepare for implementation included a 90-minute presentation to the county’s EMS advisory board by county staff and planning meetings with a public health program coordinator. Ongoing monthly costs included travel by a regional EMS coordinator to drop off naloxone kits to each EMS agency. For this, we calculated the costs in both time spent by the EMS coordinator and mileage to travel to each Regional EMS office from the Department of Health using IRS standard mileage rates ($0.65 per mile; [(23)](https://sciwheel.com/work/citation?ids=16675238&pre=&suf=&sa=0&dbf=0)). We then multiplied the ongoing monthly cost over 18 months (the amount of time the EBP was implemented) and added the 18-month cost to the one-time costs.

**S3. Sensitivity Analysis of Total EBP Cost per Community Including HEALing Communities Study-supported Staff Time, Waves 1 and 2**


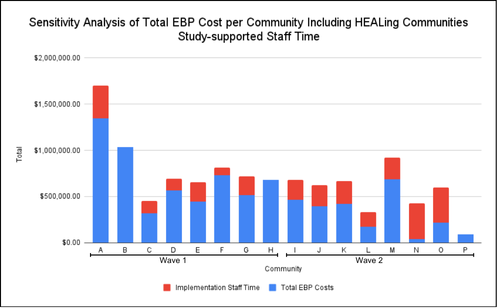


Note: EBP, evidence-based practice

**S4. Breakdown of EBPs by Costs, Waves 1 and 2**

**
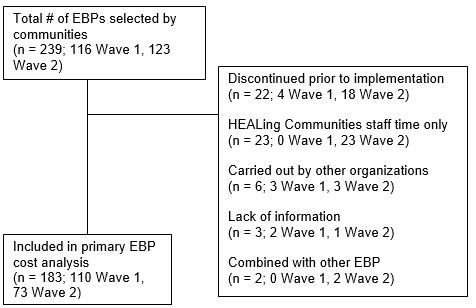
**

Note: EBP, evidence-based practice
